# Moderating Effects of Cortisol on Neural-Cognitive Association in Cognitively Normal Elderly Subjects

**DOI:** 10.3389/fnagi.2017.00163

**Published:** 2017-05-24

**Authors:** Way K. W. Lau, Mei Kei Leung, Andrew C. K. Law, Tatia M. C. Lee

**Affiliations:** ^1^Neural Dysfunction Research Laboratory, Department of Psychiatry, The University of Hong KongHong Kong, Hong Kong; ^2^Laboratory of Cognitive Affective Neuroscience, The University of Hong KongHong Kong, Hong Kong; ^3^Laboratory of Neuropsychology, The University of Hong KongHong Kong, Hong Kong; ^4^The State Key Laboratory of Brain and Cognitive Sciences, The University of Hong KongHong Kong, Hong Kong; ^5^Institute of Clinical Neuropsychology, The University of Hong KongHong Kong, Hong Kong

**Keywords:** cortisol, cognitive processing speed, brain volumes, moderation, neuroimaging

## Abstract

Cortisol homeostasis is important for healthy brain and cognitive aging. The aim of the current study is to investigate the role of serum cortisol levels in the relationship between regional brain volumes and cognitive processing speed in a group of cognitively normal elderly subjects. Forty-one healthy elderly participants were from a parallel longitudinal study. The reported data in this study reflects baseline measurements. Whole-brain anatomical scanning was performed using a 3.0 Tesla Philips Medical Systems Achieva scanner. Cognitive processing speed was assessed by the digit-symbol and symbol search tests, from the Chinese version of the Wechsler Adult Intelligence Scale—third edition (WAIS-III). Serum cortisol levels (sampled in the late morning) were measured by ELISA kits. Whole-brain regression analysis revealed that serum cortisol levels positively predicted the white matter volumes (WMV) of the right thalamus, the gray matter volumes (GMV) of the left thalamus and right cerebellar tonsil, and negatively predicted the WMV and GMV of the left middle temporal gyrus (MTG) in 41 healthy elderly participants. Furthermore, serum cortisol significantly moderated the relationship between the GMV of the left MTG and processing speed, as well as the GMV of the left thalamus and processing speed. This study provided the first piece of evidence supporting serum cortisol levels in moderating the relationship between regional brain volumes and processing speed in healthy elderly subjects. This observation enriches our understanding of the role of cortisol in brain morphology and cognitive functioning.

## Introduction

Cortisol homeostasis is important for healthy cognitive aging. Dysregulation of the hypothalamus pituitary adrenal (HPA) axis, characterized by chronically elevated basal cortisol levels, is often associated with accelerated aging (Prenderville et al., 2015) and declined cognitive functioning in the elderly (Lupien et al., 2007, 2009; Comijs et al., 2010). Understanding the role of cortisol on neuro-cognitive functions in non-clinical elderly population would bring insight into the prevention of the development of neuro-cognitive disorders, e.g., dementia. Furthermore, it could also provide the scientific basis for stress reduction programs that improved cognitive functions in healthy aging adults (e.g., Galvin et al., 2006).

The role of endogenous cortisol on cognitive function in healthy elderly subjects is relatively less studied. Thus far, only a few recent studies have demonstrated the association between cortisol and cognitive functions in healthy elderly subjects. For instance, Geerlings et al. (2015) observed that the morning cortisol levels measured 45 min after awakening were positively associated with cognitive processing speed and executive function, whereas the evening cortisol levels, sampled prior to sleep, were negatively associated with memory, cognitive processing speed, and executive function in healthy elderly subjects. Furthermore, low morning to evening cortisol ratio was shown to be associated with cognitive impairment in elderly men (Johar et al., 2015). In addition, Evans et al. (2012) also reported that executive function was positively linked to cortisol awakening response magnitude in a population of healthy older adults. These studies highlight the role of morning cortisol levels in cognitive function in elderly people. In fact, cortisol release is normally elevated in the morning to enhance energy, which has been reported to be associated with increased anticipatory states in 70 middle-class mothers (Adam and Gunnar, 2001), supporting the role of cortisol in facilitating cognitive performance. Nevertheless, there are also studies reporting no associations between diurnal cortisol levels, sampled at waking and 10 pm, and cognitive functions (e.g., Cox et al., 2015).

Cortisol has also been reported to be associated with changes in regional brain volumes. For instance, brain atrophy in bilateral hippocampal and cerebellar gray matter (GM) expressing high levels of glucocorticoid receptors (de Kloet et al., 2005; Hawrylycz et al., 2012) was observed in patients with uncontrolled Cushing’s disease, a state with high cortisol levels in the blood (Burkhardt et al., 2015). A recent meta-analysis showed that the damaging effect of high cortisol levels in Cushing’s syndrome on the human brain was irreversible (Andela et al., 2015). In healthy subjects, a brief hydrocortisone challenge was shown to significantly reduce hippocampal volume in a group of healthy adults (Brown et al., 2015). In addition, a recent study demonstrated a positive association between the morning cortisol levels and white matter volume (WMV) in the temporal, parietal and occipital lobes in a large sample of elderly subjects without dementia (Geerlings et al., 2015). In contrast, the morning plasma cortisol levels (at 9 am) have been reported to have no associations with age-related brain atrophy in a group of healthy elderly men (MacLullich et al., 2005), which may be due to the different sampling time.

There has been an increase in the number of studies reporting the relationship between cortisol, brain volumes and cognitive functions. The interaction between cortisol and its related regional brain volumes on cognitive functions is, however, less understood. In the present study, we aim to investigate the role of serum cortisol levels in the relationship between the regional brain volumes and cognitive status using moderation analyses in a group of non-depressed, cognitively normal elderly subjects. The cognitive processing speed was used to reflect the general state of cognitive functioning because it is an index sensitive to the general cognitive status (Salthouse, 1996). We hypothesized that serum cortisol significantly interacted with the regional brain volumes to predict processing speed outcomes. The findings from this study provided the first piece of evidence on the role of cortisol levels in neural-cognitive associations, which is important for monitoring the brain and cognitive aging.

## Materials and Methods

### Participants and Design

This study was approved by the Institutional Review Board of The University of Hong Kong and the Hospital Authority. All subjects gave written informed consent in accordance with the Declaration of Helsinki. Inclusion criteria were 60 years old or above, fluent in Cantonese, education level of primary one or above, normal or corrected-to-normal hearing and vision, right-handedness, scored 8 or below in the 15-item version of the Geriatric Depression Scale (GDS-15) and scored 26 or above in the Montreal Cognitive Assessment, Hong Kong Version (HK-MoCA, Wong et al., 2009). Subjects who scored 8 or below in GDS-15 were defined as normal or non-depressed (Boey and Chiu, 1998). A cutoff at 26 in HK-MoCA (after adjusted for the years of education) was chosen so as to be comparable with other international studies (Nasreddine et al., 2005). Subjects who scored 26 or above in HK-MoCA were defined as cognitively normal. Exclusion criteria were magnetic resonance imaging (MRI) incompatibility, history of brain injuries, neurological or psychiatric disease, endocrine disorders and current engagement in any psychotherapy or pharmacotherapy that may affect the functioning of autonomic and/or central nervous systems. Healthy elderly participants were from a parallel longitudinal study on meditation/relaxation training (Shao et al., 2016). Forty-one subjects who fitted in the abovementioned criteria were included in the current study. Serum cortisol sampling, assessments on cognitive processing speed and structural MRI were conducted within 3 weeks before the trainings, whereas cognitive assessments and structural MRI were conducted on the same day. The reported data in this study reflects baseline measurements.

### Processing Speed Index (PSI)

Cognitive processing speed was assessed by two subtests, namely, the digit-symbol and symbol search test, from the Chinese version of the Wechsler Adult Intelligence Scale—third edition (WAIS-III; Wechsler et al., 2002). In the digit-symbol test, different symbols were paired with a set of numbers. Participants were asked to write or draw the symbols that correspond to particular numbers. In the symbol search test, participants were asked to identify the symbols that appeared repeatedly. The cumulative raw scores of the two subtests were summed to represent Processing speed index (PSI). Higher PSI score means higher processing speed.

### Structural MRI Data Acquisition and Processing

Whole-brain anatomical scanning was performed using a 3.0 Tesla Philips Medical Systems Achieva scanner. A three-dimensional, T1-weighted, magnetization-prepared rapid-acquisition gradient-echo (MP-RAGE) sequence was used to acquire high-resolution anatomical images (164 contiguous sagittal slices, 1-mm thick, TR = 7 ms, TE = 3.2 ms, flip angle = 8°, FOV = 164 mm, matrix = 256 × 240 mm, voxel size = 1 mm^3^). The T1-images were processed using the VBM8 toolbox (Christian Gaser^1^). The default settings were used, unless otherwise specified. High-dimensional Dartel normalization approach was used (Ashburner, 2007). Modulated GM/white matter segments were generated by multiplying them with the nonlinear components derived from the normalization matrix instead of the linear components, in order to preserve the actual GM/white matter values locally and account for individual differences in global brain size. Finally, smoothing with an 8-mm full-width half-maximum (FWHM) Gaussian kernel was performed on the normalized modulated images. Serum cortisol levels were used to predict gray matter volumes (GMV) and WMV in the whole-brain. Clusters were considered significant at the combined voxel-extent threshold of an uncorrected voxel level of *p* < 0.001 and cluster extent ≥160 voxels, as determined by AlphaSim to be equivalent to *p* < 0.05, corrected for multiple comparisons. Average GMV/WMV were extracted from the significant cluster(s) using the REX toolbox (Susan Whitfield-Gabrieli^2^) for further moderation analyses.

### Blood Sampling

Blood sampling was conducted in the late morning to minimize the diurnal effect of cortisol (Touitou and Haus, 2000). The average blood sampling time was 10:52 am (standard deviation (SD) = 45 min). Peripheral blood (3 ml) was collected in vacutainers for each participant. Samples were allowed to clot for 30 min at room temperature before centrifugation at 1000× *g* for 15 min at room temperature. The upper serum layer was collected and stored at −80°C until further analysis.

### Serum Cortisol Levels

Serum cortisol levels were measured using a commercially available enzyme-linked immunosorbent assay (ELISA) kit from Enzo Life Sciences (Farmingdale, NY, USA) in duplicate per subject, according to the manufacturer’s instructions. The detection range of the kit was 0.156–10 ng/ml. The average intra-assay coefficient of variation (CV) and inter-assay CV were 5.03% and 4.74%, respectively.

### Statistical Analysis

Normality of any continuous data was examined using the Kolmogorov-Smirnov test. Serum cortisol levels were transformed by natural logarithm and the transformed values were used for relevant parametric analyses. *P*-value < 0.05 is considered statistically significant.

Moderation analyses were performed using model 1 in the PROCESS macro for SPSS developed by Hayes (2013). Briefly, the extracted brain volumes (predictors) and cortisol levels (moderators) were mean-centered. The mean-centered values together with their interaction term were entered in the linear regression model to predict processing speed indices. The effects of age, years of education and gender were adjusted (for the correlation matrix between covariates and variables of interest, please see Supplementary Table S1). The PROCESS macro is based on ordinary least squares regression and adopts a nonparametric bootstrapping procedure (1000 bootstrapped samples in this study), which gives rise to a bias-corrected confidence interval (CI) for effect size inference (Shrout and Bolger, 2002). The presence of a significant effect is denoted if zero is not included by the upper and lower bound of 95% CI (Preacher and Hayes, 2008).

## Results

### Demographic Data

We included 41 non-depressed, cognitively normal elderly men and women (age range 60–68 years old). The mean and standard deviation of serum cortisol levels were 32.34 ± 46.50 ng/mL (range = 7.95–305.77 ng/mL) in our samples, which is similar to the plasma cortisol levels reported in 95 healthy elderly men at 9 am (range = 38.48–329.10 ng/mL, age range 65–70 years old, MacLullich et al., 2005), and is much lower than the levels in Cushing’s disease patients (range = 147.05–829.31 ng/mL, Burkhardt et al., 2015). Details of the demographic data are reported in Table 1.

**Table 1:** **Demographic data of the enrolled elderly subjects (*N* = 41)**.

Variable	Mean (Standard Deviation)
Age (years)	64.71 (2.35)
Gender	
Male	15
Female	26
Education (years)	13.12 (3.77)
Serum cortisol levels (ng/mL)	32.34 (46.50)
Montreal Cognitive Assessment (MoCA) score	28.10 (1.14)
15-item version of Geriatric Depression Scale (GDS-15) score	2.90 (2.34)
Processing speed index (PSI)	92.17 (18.02)
Digit symbol	63.22 (14.57)
Symbol search	28.95 (5.14)

### Association between Serum Cortisol Levels and Regional Brain Volumes

**Figure 1:**
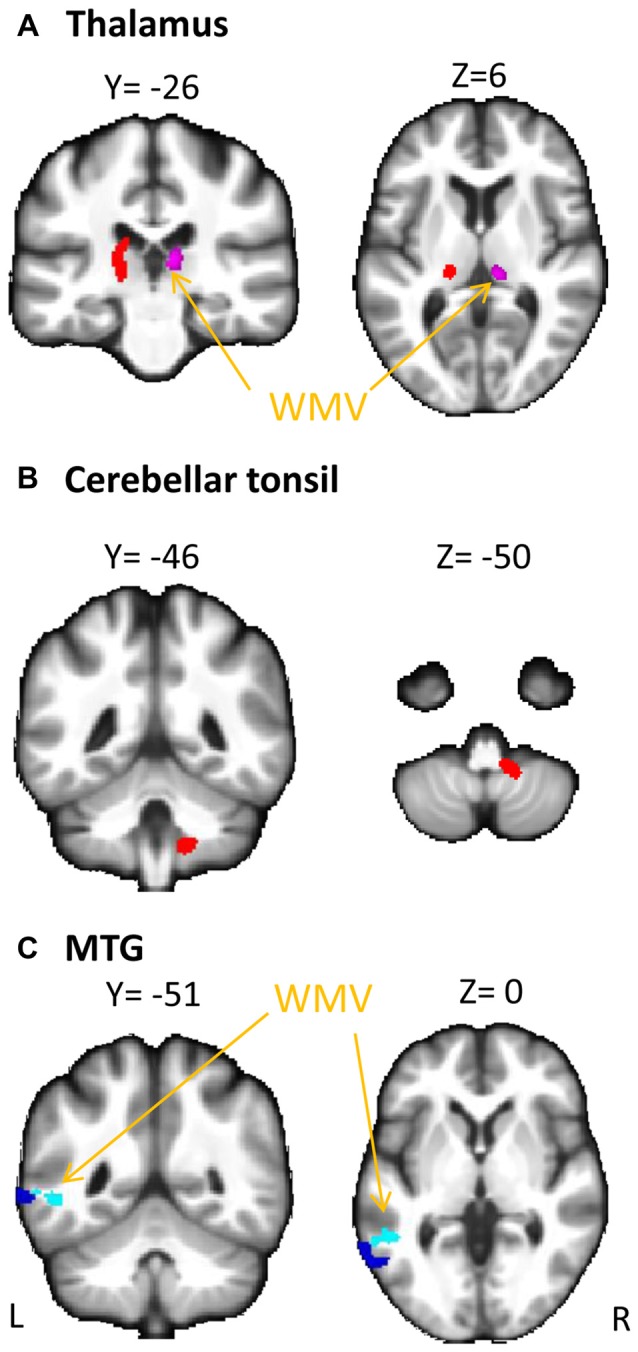
**Association between serum cortisol levels and gray matter volume (GMV) and white matter volume (WMV)**. Serum cortisol levels (transformed by natural logarithm) positively correlated with **(A)** GMV of the left thalamus (red clusters) and WMV of the right thalamus (violet clusters with arrows) and **(B)** GMV of the cerebellar tonsil (red clusters), and negatively correlated with **(C)** GMV (blue clusters) and WMV (light blue clusters with arrows) of the middle temporal gyrus (MTG). L, left; R, right.

Whole-brain regression analysis revealed that serum cortisol levels positively predicted the WMV of the right thalamus, and negatively predicted the WMV of the left middle temporal gyrus (MTG). In addition, serum cortisol levels also positively predicted the GMV of the left thalamus and right cerebellar tonsil, and negatively predicted the GMV of the left MTG (Figure 1 and Table 2). The brain volumes of these regions were extracted to study the moderating role of serum cortisol levels in the association between processing speed and the brain volumes.

**Table 2:** **Regression with whole-brain GMV and WMV predicted by serum cortisol level**.

	Association with serum cortisol level	Brain region	Peak coordinates in MNI space			*t-* value	Cluster size
			*X*	*Y*	*Z*
WMV	Positive	Right thalamus	10	−25	9	4.90	201*
	Negative	Left middle temporal gyrus	−50	−48	−2	3.87	203*
			−58	−46	3	3.83	
			−57	−57	−2	3.59	
GMV	Positive	Left thalamus	−15	−27	16	4.48	241*
			−18	−25	3	3.99	
	Positive	Right cerebellar tonsil	12	−43	−51	4.54	186*
	Negative	Left middle temporal gyrus	−57	−60	4	4.36	491*
			−68	−54	−3	3.75	
			−54	−61	15	3.73	

Note: GMV, Gray Matter Volume; MNI, Montreal Neurological Institute; WMV, White Matter Volume. *Corrected p < 0.05 as defined by uncorrected voxel-level threshold p < 0.001 and cluster-level threshold ≥ 160 voxels.

### Moderating Role of Serum Cortisol in Neural-PSI Association

#### WMV of the Right Thalamus

The interaction effect between serum cortisol and WMV of the right thalamus was not significant (*t* = −1.917, *p* = 0.064) after adjusted for the effect of age, years of education and gender. Neither cortisol nor WMV of the right thalamus had a significant effect on PSI (*p* > 0.05).

#### WMV of the Left MTG

The interaction effect between serum cortisol and WMV of the left MTG was not significant (*t* = 0.698, *p* = 0.490) after adjusted for the effect of age, years of education and gender. Neither cortisol nor WMV of the left MTG had a significant effect on PSI (*p* > 0.05).

#### GMV of the Right Cerebellar Tonsil

The interaction effect between serum cortisol and the GMV of the right cerebellar tonsil was not significant (*t* = −1.608, *p* = 0.117) after adjusted for the effect of age, years of education and gender. Neither cortisol nor the GMV of the right cerebellar tonsil had a significant effect on PSI (*p* > 0.05).

#### GMV of the Left MTG

**Figure 2:**
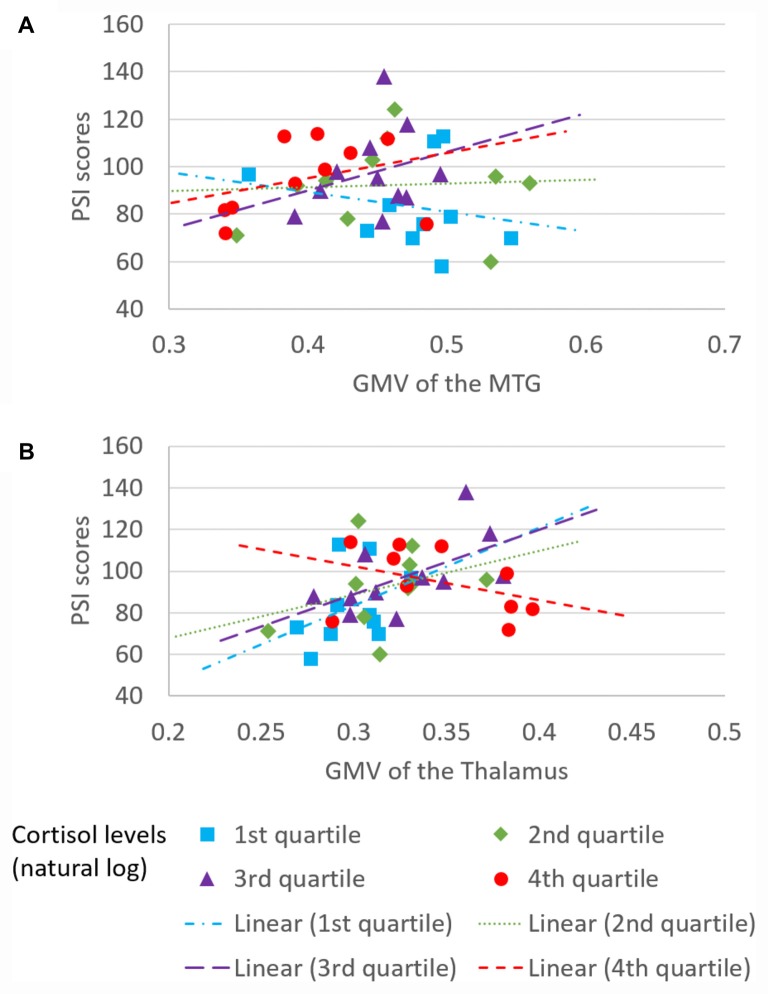
**Moderating effect of morning serum cortisol levels on neural-cognitive associations**. Serum cortisol levels moderated the relationship between processing speed index (PSI) and **(A)** GMV of the left MTG and **(B)** GMV of the left thalamus.

The overall regression model in predicting PSI using serum cortisol levels, the GMV of the left MTG and their interaction term as independent variables was significant after controlled for the effects of age, years of education and gender (*F*_(6,34)_ = 4.100, *p* = 0.0034), which accounted for 42.00% of variance in explaining PSI. The interaction term of serum cortisol levels and the GMV of the left MTG was a significant predictor of PSI (unstandardized coefficient = 118.430, standard error = 57.875, *p* = 0.0485). This indicated that serum cortisol levels moderated the relation between the GMV of the left MTG and PSI. Increased levels of serum cortisol reversed the effect of the GMV of the left MTG on PSI scores (Figure 2A). Neither serum cortisol nor the GMV of the left MTG had a significant effect on PSI (*p* > 0.05), suggesting a complete moderation. In the sub-scale analysis, serum cortisol levels were found to significantly moderate the relationship between the GMV of the left MTG and digit symbol scores, but not symbol search scores (Table 3).

**Table 3:** **Moderating role of serum cortisol in the association between the regional GMV and processing speed index (PSI) subscales**.

	Unstandardized coefficient	Standard error	*t*-value	*p*-value
**GMV of the left MTG**				
*PSI*				
Serum cortisol levels	9.897	5.166	1.916	0.064
GMV of the left MTG	36.585	56.495	0.648	0.522
Interaction term	118.430	57.875	2.046	**0.049**
*Digit symbol*				
Serum cortisol levels	6.500	4.302	1.511	0.140
GMV of the left MTG	22.143	47.041	0.471	0.641
Interaction term	99.607	48.190	2.067	**0.046**
*Symbol search*				
Serum cortisol levels	3.396	1.571	2.162	**0.038**
GMV of the left MTG	14.442	17.176	0.841	0.406
Interaction term	18.823	17.596	1.070	0.292
**GMV of the left thalamus**				
*PSI*				
Serum cortisol levels	4.319	4.908	0.880	0.385
GMV of the left thalamus	167.484	81.508	2.055	**0.048**
Interaction term	−204.201	93.475	−2.185	**0.036**
*Digit symbol*				
Serum cortisol levels	1.563	4.194	0.373	0.712
GMV of the left thalamus	128.720	69.657	1.848	0.073
Interaction term	−139.901	79.884	−1.751	0.089
*Symbol search*				
Serum cortisol levels	2.756	1.452	1.898	0.066
GMV of the left thalamus	38.764	24.120	1.607	0.117
Interaction term	−64.300	27.661	−2.325	**0.026**

*Note: GMV, Gray matter volume; PSI, Processing speed index. The effect of age, years of education and gender were adjusted. *P*-values < 0.05 were bold*.

#### GMV of the Left Thalamus

The overall regression model in predicting PSI using serum cortisol levels, the GMV of the left thalamus and their interaction term as independent variables was significant after controlled for the effects of age, years of education and gender (*F*_(6,34)_ = 4.946, *p* = 0.0010), which accounted for 46.61% of variance in explaining PSI. The GMV of the left thalamus, but not serum cortisol, had a significant effect on PSI (unstandardized coefficient = 167.484, standard error = 81.508, *p* = 0.0476). The interaction term of serum cortisol levels and the GMV of the left thalamus was a significant predictor of PSI (unstandardized coefficient = −204.201, standard error = 93.475, *p* = 0.0359), indicating that serum cortisol levels also moderated the relationship between the GMV of the left thalamus and PSI. Increased levels of serum cortisol reversed the effect of the GMV of the left thalamus on PSI scores (Figure 2B). In sub-scale analysis, serum cortisol levels were found to significantly moderate the relationship between the GMV of the left thalamus and symbol search scores, but not digit symbol scores (Table 3).

## Discussion

The current study applied moderation analyses to understand the role of serum cortisol levels in the relationship between regional brain volumes and cognitive processing speed. Our study emphasized on the moderating role of serum cortisol levels in the neural-cognitive association. We also demonstrated associations between serum cortisol levels and regional brain volumes, including both the GMV and WMV, which corroborates some of the previous observations from the literature. Our findings support the importance of cortisol homeostasis in monitoring morphological and cognitive changes seen in healthy elderly people.

In a previous study on a large sample of elderly subjects without dementia, the morning saliva cortisol levels (sampled 45 min after awakening) were shown to be positively associated with the WMV in different lobes of the brain as well as processing speed and executive function, but not with the GMV (Geerlings et al., 2015). In this study, using a whole brain voxel wise approach, we found that the late morning serum cortisol levels were positively associated with the WMV of the right thalamus, the GMV of the left thalamus and right cerebellar tonsil, and were negatively associated with the GMV and WMV of the left MTG (Figure 1, Table 2). The differences in the findings between Geerlings’s and our study may be due to the use of average volume of bilateral brain regions in their study, which may render the investigation of the association between morning cortisol levels and GM less sensitive, especially if the effect is laterality-specific. Also, the difference in cortisol sampling time between the two studies could potentially affect the outcomes. The brain regions that we found to be correlated with serum cortisol levels express glucocorticoid receptors in humans (Sarrieau et al., 1988; Yu et al., 2002) and have been reported to be associated with cognitive functions. For example, higher high-gamma power in the left thalamus was associated with better processing speed among healthy elderly adults using magnetoencephalogram (Akimoto et al., 2014). Hypometabolism in the right cerebellar tonsil has been reported in illiterate elderly subjects with poorer cognitive functions compared to age- and gender-matched literate subjects (Kwon et al., 2012). The role of cerebellum in controlling incoming working memory information was also demonstrated in patients with cerebellar ischemic stroke (Baier et al., 2014). The positive associations between serum cortisol levels and these regions may imply a facilitating role of cortisol in cognitive functions via these brain regions. On the other hand, atrophy of the bilateral thalamus and left MTG were consistently found in patients with mild cognitive impairment or dementia (Yang et al., 2012a,b). The negative association between serum cortisol levels and regional brain volumes of the left MTG indicates a region-specific response to cortisol in the MTG, which is different from the cortisol response in the thalamus and cerebellum. This observation requires more future studies to confirm. Taken together, our findings suggest the close relationship between the morning serum cortisol levels and the WMV/GMV of brain regions that are important for cognitive functions in cognitively normal elderly people.

We further investigated the moderating role of serum cortisol levels in the neural-cognitive association that involved the abovementioned brain regions. From the moderation analyses, we found a significant moderating role of the serum cortisol levels in the relationship between the GMV of the left MTG and PSI. Increased cortisol levels changed the relationship between the GMV of the left MTG and PSI from negative to positive (Figure 2A), suggesting that cortisol facilitates the left MTG in performing the cognitive task. The MTG is associated with many different functions, such as language processing (Mirz et al., 1999), observation of motion (Rizzolatti et al., 1996), deductive reasoning (Goel et al., 1998) and dynamic facial expressions (Sato et al., 2012). Recently, different parts of the MTG have been reported to functionally and anatomically connect with other brain regions that are involved in memory retrieval (e.g., hippocampus) and executive controls (e.g., inferior frontal gyrus; Xu et al., 2015), which is believed to have substantial influence on processing speed (Cepeda et al., 2013). Interestingly, the moderating effect of serum cortisol levels was specific to the digit symbol sub-scales of PSI, of which the performance is significantly contributed by memory functions assessed by incidental learning and Wechsler Memory Scale III (immediate and delayed recall for visual and auditory domains; Joy et al., 2004). Our findings suggest that the effect of the interaction between cortisol and the MTG on processing speed involves a memory component. Unfortunately, memory domains were not captured in the current study. Therefore, such speculation requires further studies to confirm.

We also found a significant moderating role of the serum cortisol levels in the association between the GMV of the left thalamus and the PSI. In contrast to the finding in the MTG, positive associations between the GMV of the left thalamus and PSI were observed in subjects with low to medium serum cortisol levels (from the 1st to the 3rd quartile, Figure 2B), whereas a negative association between the GMV of the left thalamus and PSI was observed in subjects with high serum cortisol levels (the 4th quartile, Figure 2B). Furthermore, such moderating effect was specific to the other sub-scale of PSI—the symbol search task—that does not rely on memory as much as the digit symbol task. One speculation for this observation is that glucocorticoid receptors are more densely expressed in the thalamus. Within the optimal range of cortisol, the bigger volume of the thalamus (plausibly with more glucocorticoid receptors) could bind more cortisol, which subsequently influences perceptual detection thresholds. This can then enhance focused attention on the perceived stimuli and excludes irrelevant stimuli (Fehm-Wolfsdorf and Nagel, 1996; Erickson et al., 2003), resulting in better processing speed. In contrast, excess cortisol can down-regulate glucocorticoid receptors in the brain (Sapolsky and McEwen, 1985), which reduces the regional brain responsiveness to cortisol stimulations, and hence, minimizes the effect of cortisol on processing speed. Specifically, the thalamic region that we found to be associated with cortisol levels was located approximately at the lateral thalamic nuclei that were functionally related to spatial attentional weighting, selective attentional control and visual perceptual processing speed (Kraft et al., 2015). We, therefore, speculate that the cortisol-thalamic interaction effect on cognitive processing speed may be mediated through visual attentional processing. This speculation requires more empirical studies to confirm. Consistent with the literature, our findings further corroborate the non-linear relationship between cortisol levels and cognitive function (for review see Belanoff et al., 2001) by interacting with the regional brain volumes. The differential interaction effects between serum cortisol levels, the MTG and the thalamus might be due to the regional-specific reduction in glucocorticoid receptors in the brain during aging, which requires more empirical studies to confirm.

## Limitations

There are several limitations in the current study. First, interpretations on results are limited by the current study design. Although we adopted moderation models, the causality among those markers of interest cannot be confirmed without a longitudinal design. Second, serum cortisol was sampled only once in the late morning (~11 am). The effect of cortisol awakening response and the diurnal effect of cortisol on the regional brain volumes and cognitive function cannot be deduced in the current study. Third, the absence of information on other domains of cognitive ability or other brain imaging modalities would limit our interpretations on the results. Furthermore, cautions should be taken when interpreting the results due to the limited sample size in the current study. Last, blood sampling was done beyond the morning period (12–1 pm) in four subjects. Nonetheless, all significant findings maintained after these four subjects were removed in a sensitivity test, suggesting the effect of this small delay on our findings is minimal. Future studies that directly manipulate the level of cortisol with a greater sample size could provide a more direct impact of this stress marker on the brain and cognitive aging.

## Conclusion

To conclude, our study provided the first piece of evidence to support the moderating effects of serum cortisol levels on the relationship between regional brain volumes and processing speed. Our findings provide insight into the role of cortisol in modulating brain morphology and cognitive functions in healthy elderly subjects.

## Author Contributions

TMCL and ACKL conceived and designed the study. TMCL supervised data acquisition. WKWL and MKL analyzed the data. WKWL interpreted the data and drafted the article. All authors revised the article critically for important intellectual content and have approved the final article.

## Conflict of Interest Statement

The authors declare that the research was conducted in the absence of any commercial or financial relationships that could be construed as a potential conflict of interest.

## Footnotes

^1^http://dbm.neuro.uni-jena.de/vbm/download/

^2^http://web.mit.edu/swg/software.htm
