# Supplementary material for: Moderating Effects of Cortisol on Neural-Cognitive Association in Cognitively Normal Elderly Subjects
Source: Front Aging Neurosci. 2017 May 24;9:163. doi: 10.3389/fnagi.2017.00163 (PMC5443153; doi:10.3389/fnagi.2017.00163)
Supplement: Supplementary file 1 [file Table_1.docx]

**Appendix**

**Supplementary Table 1: Correlation matrix between the covariates (age, gender and education) and the variables of interest**

| Variables | (1) | (2) | (3) | (4) | (5) | (6) | (7) | (8) | (9) | (10) | (11) |
| --- | --- | --- | --- | --- | --- | --- | --- | --- | --- | --- | --- |
| (1) Age |  |  |  |  |  |  |  |  |  |  |  |
| (2) Gender (male=1, female=2) | 0.162 |  |  |  |  |  |  |  |  |  |  |
| (3) Education | -0.225 | -0.356* |  |  |  |  |  |  |  |  |  |
| (4) GMV of the cerebellar tonsil R | 0.036 | -0.391* | 0.007 |  |  |  |  |  |  |  |  |
| (5) GMV of the thalamus L | -0.068 | -0.319* | 0.217 | 0.369* |  |  |  |  |  |  |  |
| (6) GMV of the MTG L | -0.027 | 0.544** | -0.297 | -0.112 | -0.262 |  |  |  |  |  |  |
| (7) WMV of the thalamus R | 0.199 | -0.318* | 0.072 | 0.219 | 0.571** | -0.336 |  |  |  |  |  |
| (8) WMV of the MTG L | 0.070 | 0.452** | -0.143 | -0.391* | -0.219 | 0.448** | -0.181 |  |  |  |  |
| (9) Serum cortisol levels | -0.015 | -0.502** | 0.230 | 0.574** | 0.579** | -0.584** | 0.578** | -0.540** |  |  |  |
| (10) Digit symbol scores | -0.512** | -0.069 | 0.299 | -0.030 | 0.251 | -0.006 | -0.128 | -0.231 | 0.067 |  |  |
| (11) Symbol search scores | -0.477** | -0.217 | 0.171 | 0.025 | 0.326* | -0.080 | -0.055 | -0.348* | 0.292 | 0.574** |  |
| (12) PSI scores | -0.550** | -0.118 | 0.291 | -0.017 | 0.296 | -0.028 | -0.119 | -0.286 | 0.137 | 0.972** | 0.750** |

Note: GMV=Gray Matter Volume, L=Left, MTG=Middle Temporal Gyrus, PSI=Processing Speed Index, R=Right, WMV=White Matter Volume.

**p*<0.05, ***p*<0.01 (Pearson’s correlation)
